# Flexible Bronchoscopy and Non-Small-Cell Lung Cancer Staging: A Narrative Review of Modern Techniques for Optimized Clinical Decision-Making

**DOI:** 10.3390/jcm14165773

**Published:** 2025-08-15

**Authors:** Simona-Maria Roșu, Denisa Maria Mitroi, Oana Maria Catană, Viorel Biciușcă, Sorina Ionelia Stan, Beatrice Mahler, Oana-Andreea Parliteanu, Adina Andreea Mirea, Mara Amalia Bălteanu

**Affiliations:** 1 Doctoral School, University of Medicine and Pharmacy, 200349 Craiova, Romania; simona.vulpe1995@gmail.com (S.-M.R.); denisa_maria2@yahoo.com (D.M.M.); oana_cattana@yahoo.com (O.M.C.); 2 Department of Pneumology, “Marius Nasta” Institute for Pneumology, 050159 Bucharest, Romania; beatrice.mahler@umfcd.ro (B.M.); mara.balteanu@prof.utm.ro (M.A.B.); 3 Department of Internal Medicine, Faculty of Medicine, University of Medicine and Pharmacy of Craiova, 200349 Craiova, Romania; sorina_stan@icloud.com; 4 Department of Pneumology Faculty of Medicine, University of Medicine and Pharmacy Carol Davila of Bucharest, 020021 Bucharest, Romania; 5 Department of Pathophysiology, Faculty of Medicine, “Titu Maiorescu” University, 040051 Bucharest, Romania; 6 Department of Oral-Dental Prevention, University of Medicine and Pharmacy, 200349 Craiova, Romania; adinaturcu14@yahoo.com; 7 Department of Pneumology, Faculty of Medicine, “Titu Maiorescu” University, 040051 Bucharest, Romania

**Keywords:** flexible bronchoscopy, non-small-cell lung cancer, diagnosis, staging, EBUS-TBNA

## Abstract

Non-small-cell lung cancer (NSCLC) is a widespread and aggressive form of cancer, and in cases of its occurrence, accurate diagnosis and precise staging play a crucial role in determining treatment and estimating prognosis. Flexible bronchoscopy (FB) is a minimally invasive method used to assess the local and regional extent of the disease. FB facilitates the identification of endobronchial lesions and the collection of biopsy samples for histopathological diagnosis. It also enables the evaluation of regional lymph node involvement via advanced techniques such as endobronchial ultrasound with fine-needle aspiration (EBUS-TBNA). This method has high sensitivity and specificity, reducing the need for more invasive interventions like mediastinoscopy. The integration of endobronchial ultrasound (EBUS) has revolutionized NSCLC staging by providing detailed images and guiding biopsies of suspicious lymph nodes. Additionally, FB is valuable in staging the extent of primary tumor growth, providing critical information about the invasion of adjacent structures. In conclusion, FB, supported by advanced technologies, is important for the staging of NSCLC, improving medical practice and patient prognosis.

## 1. Introduction

In 2020, lung cancer (LC) exceeded breast cancer in cancer mortality frequency among men in Europe, and it also accounted for about 12% of all new cancer diagnoses [1]. In Romania, LC constitutes a considerable public health issue, representing approximately 20% of deaths from cancer. Tragically, only 10% of the LC Romanian patients survived after the first 5 years, primarily due to late-stage detection [2]. NSCLC, which encompasses subtypes such as adenocarcinoma, squamous cell carcinoma, and large cell carcinoma, comprises approximately 85% of LC cases. However, less than one in three NSCLC patients are identified in the early stages, when the disease is still potentially treatable [3]. Given the overwhelming financial burden of lung cancer treatment, with the U.S. spending USD 1.5 billion on care in 2019, timely and accurate diagnosis is crucial both clinically and economically [4]. FB has become an essential, minimally invasive procedure for diagnosing and assessing lung cancer, offering vital insight into the local and regional extent of the disease. One of the primary challenges of this technique is the difficulty in obtaining biopsy samples from peripheral lesions using auxiliary instruments. Once these instruments pass the bronchoscope’s tip, directing them to the target area becomes problematic. Locating lesions under fluoroscopy can be difficult, leading to a preference for alternative guidance techniques such as CT-guided bronchoscopy and EBUS. EBUS-TBNA has gained increasing use as a first-line diagnostic approach for centrally located lesions that lack endobronchial visibility and/or present adenopathy. This minimally invasive technique aids in both diagnosis and disease staging, and it is now recommended when adenopathy is detected on CT scans or when lymph nodes exhibit fluorodeoxyglucose (FDG) uptake on PET-CT [5]. The purpose of this article is to emphasize the importance of bronchoscopy in clinical practice, offering evidence that supports informed clinical decisions and contributes to better patient outcomes, ultimately advancing pulmonary oncology.

## 2. Methodology and Search Strategy

A structured and methodologically transparent literature review was performed to identify relevant studies on the use of FB, EBUS-TBNA, and other diagnostic modalities for staging of NSCLC. The databases searched included PubMed, Scopus, and Web of Science, covering publications from 2010 to 2024, and a total of 32 articles were included in the final analysis.

The search strategy combined the following keywords and Boolean operators: (“NSCLC staging” OR “lung cancer staging”) AND (“flexible bronchoscopy” OR “EBUS-TBNA” OR “BAL” OR “mediastinoscopy” OR “PET/CT” OR “AI” OR “electromagnetic navigation bronchoscopy (ENB)”) AND (“diagnostic accuracy” OR “sensitivity” OR “specificity”). Inclusion criteria encompassed original research articles, meta-analyses, and systematic reviews published in English, involving human subjects, and reporting diagnostic performance metrics such as sensitivity, specificity, positive and negative predictive values, or overall diagnostic yield. Only peer-reviewed studies were considered. Case reports or case series with fewer than 10 patients, the non-peer-reviewed literature, preclinical studies, animal models, and abstracts or editorials lacking original data were excluded from our analysis.

## 3. Flexible Bronchoscopy and Adjunct Techniques: The Role of BAL and Bronchial Brushing in NSCLC Evaluation

**Figure 1:**
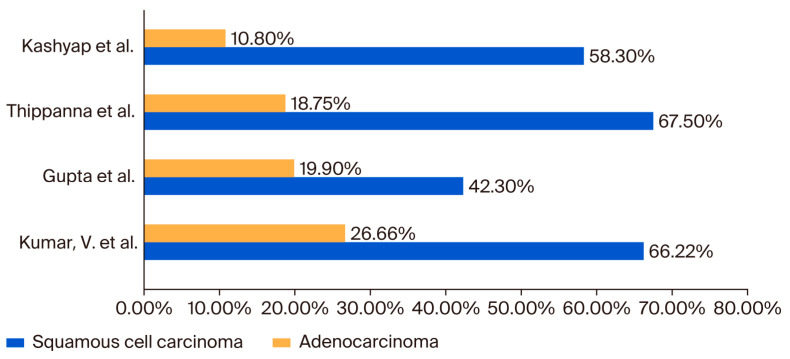
Hystopathological lung cancer types identified via flexible bronchoscopy in published studies. Kumar V. et al. (2017) [14], Gupta et al. (1998) [17], Thippanna et al. (1999) [18], Kashyap et al. (2003) [19].

FB is indispensable for identifying endobronchial lesions, staging lung cancer, and assessing invasion of the trachea, bronchi, and adjacent structures to guide treatment. It helps differentiate between resectable and unresectable tumors, thereby influencing therapeutic decisions, and it is a minimally invasive method for diagnosing LC, considered the safest and most accessible technique for obtaining biopsy material, and the gold standard in diagnosing invasive and preinvasive malignant lesions of the airways. This technique is performed using a flexible fiber-optic bronchoscope introduced through the mouth or nose, allowing for direct visualization of the tracheobronchial tree. It is primarily employed for centrally located tumors, visible lesions, or when symptoms such as hemoptysis or airway obstruction suggest endobronchial involvement. The procedure is generally performed under local anesthesia and moderate sedation. FB employs various tools depending on lesion visibility and location. Biopsy forceps are typically used to obtain tissue samples from visible endobronchial tumors and involve taking 3–5 samples to improve diagnostic accuracy. This method has become the recommended approach for all patients with suspected lung cancer, presenting a sensitivity of approximately 88% for centrally located lesions and about 78% for peripheral lesions (ranging from 36% to 88% depending on the technique used). Additionally, FB enables proper surgical planning by assessing the surface, location, tumor extent, vocal cord mobility, and lumen of the airways [6]. Patients diagnosed at an early stage of the disease have a 5-year survival rate greater than 70%, confirming that early LC detection is essential for improving the odds of survival. Given that the early detection of lung cancer, followed by prompt initiation of treatment, can improve outcomes, considerable efforts have been made to overcome the limitations of FB in white light. Innovative bronchoscopic techniques, such as autofluorescence bronchoscopy (AFB) and narrow-band imaging (NBI) bronchoscopy, have been developed to detect preinvasive malignant lesions. Wang et al. demonstrated that AFB had significantly higher sensitivity in detecting malignant and premalignant lesions compared to white light bronchoscopy (WLB). Early detection of lung cancer is indeed possible. NBI is a variant of AFB that enhances specificity for diagnosing lung cancer. A 2015 meta-analysis which encompassed eight studies found that NBI had a sensitivity of 80% and specificity of 84%. When NBI was combined with AFB, the sensitivity and specificity increased to 86% and 75%, respectively [7]. FB plays an important role in staging tumors by identifying the local extension of the primary lesion (the T component of the TNM staging system). This provides essential information regarding the invasion of the trachea, main bronchi, or adjacent structures, which is crucial for determining a patient’s suitability for surgical intervention or curative radiotherapy. The ninth edition of the TNM staging system stated that T1 tumors do not involve the primary or lobar bronchi. T2 tumors include lesions that invade a main bronchus, regardless of their proximity to the carina. Tumors of any size that invade the carina are classified as T4 [8]. All of these characteristics, which highlight the degree of tissue invasion and the involvement of central airways, are essential for surgical planning; the invasion of the origin of the lobar or main bronchus may require sleeve resection or pneumonectomy. In the LC approach, it is also crucial to distinguish between resectable and unresectable lesions, given that unresectable tumors are managed with chemotherapy, immunotherapy, and/or radiotherapy only. When lesions are less well defined or localized to the peripheral airways, bronchial brushings and bronchoalveolar lavage (BAL) are more appropriate for cytological sampling. Exfoliative cytology techniques are useful for collecting cells from mucosal surfaces, providing diagnostic information about preinvasive or incipient malignant changes. These tools, when appropriately combined, increase diagnostic accuracy and support optimal clinical decision-making [9]. BAL is a minimally invasive technique used during bronchoscopy that plays an important role in the evaluation and diagnosis of patients with suspected peripheral NSCLC. Combining FB with BAL is particularly useful when lung lesions are inaccessible for direct biopsy. BAL represents a complementary method within the diagnostic algorithm of NSCLC and involves the instillation of sterile saline solution into the distal airways, followed after 10–15 min by aspiration of the fluid, which is subsequently analyzed through biochemical, cytological, and microbiological examinations [10]. BAL can contribute to the diagnosis of NSCLC by detecting malignant cells in bronchial secretions, especially in patients with visible endobronchial lesions [11]. Cytological analysis of the retrieved fluid can identify malignant cells, determine histologic subtype, and sometimes even detect actionable molecular mutations relevant for targeted therapies (e.g., EGFR, KRAS, ALK) through a polymerase chain reaction (PCR) analysis of cellular DNA collected from the BAL supernatant [12]. In addition to its diagnostic value, BAL is useful for excluding other conditions that may mimic lung cancer, such as infections or inflammatory diseases, thus supporting differential diagnosis in NSCLC. The sensitivity of BAL in detecting and analyzing neoplastic cells is variable and depends on factors such as tumor location, the degree of cellular exfoliation, and the expertise of the cytopathology laboratory [13]. Bronchial brushing is a technique in which the bronchial wall is brushed with flexible brushes guided through a fiber-optic bronchoscope under visual or radiographic control, aimed at exfoliating and collecting cells from the bronchial mucosa. It is advantageous in cases of minimal changes in the bronchial mucosa that cannot be biopsied, such as preinvasive lesions [11,12]. The histopathological subtypes of NSCLC include adenocarcinoma, squamous cell carcinoma, and large-cell carcinoma. Vishak Acharya K et al. highlighted only 11 cases out of 156 where the diagnosis was established through lavage, with a very low sensitivity of 7%. When comparing cytology by brushing with biopsy, brushing showed a sensitivity of 55.88% and a specificity of 55.56%, suggesting a better approach by combining the two techniques. Additionally, brushing had a superior yield in cases of minimally modified mucosa, with a 50% yield, increasing to 70% in infiltrative lesions. Brushing can be preferred over biopsy with forceps in patients with a high risk of bleeding. Moreover, the friable, necrotic superficial tissue obtained via biopsy is often histopathologically inconclusive [13]. Transbronchial biopsy, commonly used for tumors located peripherally, can be performed either blindly or with fluoroscopic guidance. In their study, Kumar V et al. found that for tumors not visible through bronchoscopy, the diagnostic yield was 46.15% for BAL, 76.92% for brushing, and 84.61% for transbronchial biopsy [14]. Liam CK et al. noted that for tumors visible through bronchoscopy, the diagnostic yields were 28.3% for BAL, 77.5% for endobronchial biopsy, and 53.7% for brushing [15]. Research suggests a higher rate of squamous cell carcinoma diagnosis through biopsy compared to adenocarcinoma, likely due to the central localization of squamous cell carcinoma, which makes it easier to access [16]. Kumar V et al. found that 66.22% of patients in their study were diagnosed with squamous cell carcinoma, while 26.66% were diagnosed with adenocarcinoma. Most studies conducted in India suggest a higher prevalence of squamous cell carcinoma [14]. Gupta et al. found that 42.3% of cases were squamous cell carcinoma, while only 19.9% were adenocarcinoma [17]. Thippanna et al. identified that 67.5% of patients had squamous cell carcinoma compared to 18.75% with adenocarcinoma [18]. Kashyap et al. also highlighted that 58.3% of cases were squamous cell carcinoma and 10.8% of cases were adenocarcinoma in their study group [19]. Considering the studies presented above, a preliminary conclusion is that the use of the described bronchoscopic techniques and methods results in a superior identification rate for squamous cell carcinoma (Figure 1) compared to adenocarcinoma in lung cancer can be made. Additionally, the primary methods used for sample collection required for histopathological examination, with superior diagnostic yields, are endobronchial biopsy and brushing.

Even though FB is generally considered a safe procedure, it can be associated with several complications. These are usually rare, especially when the procedure is performed by experienced medical personnel in a controlled setting. However, complications can occur, especially during blind maneuvers such as transbronchial biopsy, and must be promptly recognized and managed. One of the most common complications is iatrogenic hemorrhage, which can range from minor, self-limiting bleeding to significant episodes that require hemostatic intervention. The risk of hemorrhage increases in the presence of hypervascularized lesions, centrally located bronchial tumors with vascular invasion, or in patients with coagulation disorders [20]. Another potential complication is pneumothorax, especially after transbronchial biopsy of peripherally located lesions, where there is an increased risk of pulmonary parenchymal perforation and air leakage into the pleural space. The incidence of pneumothorax varies depending on the size and extent of the lesion but the experience of the operator is also important [21]. Post-procedural respiratory infections, although uncommon, may occur, particularly in the context of immunosuppression, pre-existing chronic lung disease, or repeated invasive exploration of the bronchial tree [22]. Other adverse reactions encountered with FB include bronchospasm, transient hypoxemia, and side effects related to sedatives or local anesthetics used during the procedure. Therefore, careful pre-procedural risk assessment and close monitoring during and after bronchoscopy are essential for the prevention and prompt treatment of these adverse events.

## 4. Modern NSCLC Diagnostic Methodology

In contemporary practice, new technologies such as EBUS-TBNA have been introduced that feature combined imaging methods. This technique is also useful for identifying the involvement of mediastinal and hilar lymph nodes, which is necessary for staging NSCLC, along with obtaining biopsy material to confirm the histopathological diagnosis [23]. EBUS-TBNA is a procedure that combines flexible bronchoscopy with real-time ultrasound imaging and allows for the sampling of mediastinal and hilar lymph nodes. This technique constitutes a fundamental element in the staging of NSCLC, most especially for diagnosing N2 and N3 lymph node involvement. Patient preparation is performed according to institutional protocol, typically involving either conscious sedation or general anesthesia. A convex-probe bronchoscope and a 7.5 MHz ultrasound transducer are typically used. Lymph nodes are selected during the procedure for aspiration based on imaging criteria such as a size greater than 1 cm, rounded shape, absence of adipose hilum, heterogeneous echogenicity, and FDG avidity on PET-CT. Generally, a 21 G or 22 G aspiration needle is used, and for adequate tissue acquisition, a minimum of three round-trip passes of the needle per target lymph node are recommended. The application of the rapid on-site evaluation (ROSE) method, in which cytological specimens are immediately evaluated by a pathologist or trained technician, can significantly improve diagnostic accuracy and procedure yield. Doppler ultrasound is used to ensure the safety of the procedure by identifying and avoiding vascular structures before needle insertion. The puncture is generally performed at an angle of 20 to 25 degrees to the ultrasound probe, which allows for precise navigation and efficient penetration of the target node [24]. The last ten years have seen a sharp rise in the use of this minimally invasive procedure. EBUS-TBNA devices are present in about 85% of the critical care and pulmonary services that take part in different studies. Consequently, the rising use of this bronchoscopy technique for lung cancer detection and staging is becoming more and more popular. EBUS, which provides real-time pictures of mediastinal structures and enables precise navigation of the biopsy needle to suspicious lymph nodes, has transformed the staging of NSCLC by its incorporation into FB [25]. Also, EBUS-TBNA has a specificity of over 90% and a sensitivity of over 85%, which minimizes the need for more prevalent and invasive techniques like mediastinoscopy. The American Thoracic Society (ATS), European Respiratory Society (ERS), European Society of Medical Oncology (ESMO), and the American College of Chest Physicians (ACCP) have issued specific guidelines for performing invasive biopsy procedures on mediastinal adenopathy in NSCLC staging. In the latest third edition of the ACCP lung cancer guidelines, EBUS-TBNA is recommended as the first-line approach. As advanced-stage NSCLC is generally linked to a significant drop in survival rates, precise staging is very important for ensuring that patients receive the most appropriate treatment. EBUS-TBNA has also proven to be a safe technique, with minimal complications that may occur post-procedure [26]. A comprehensive systematic review including more than 1500 patients found only minor complications such as agitation, coughing, and mild bleeding at the puncture site [27]. Prospective studies have reported a complication rate of about 1%, although rare occurrences like mediastinitis, pericarditis, and even death have been noted. Despite the overall safety profile of this bronchoscopy technique, the primary risk associated with the procedure is incorrect staging [28].

**Figure 2:**
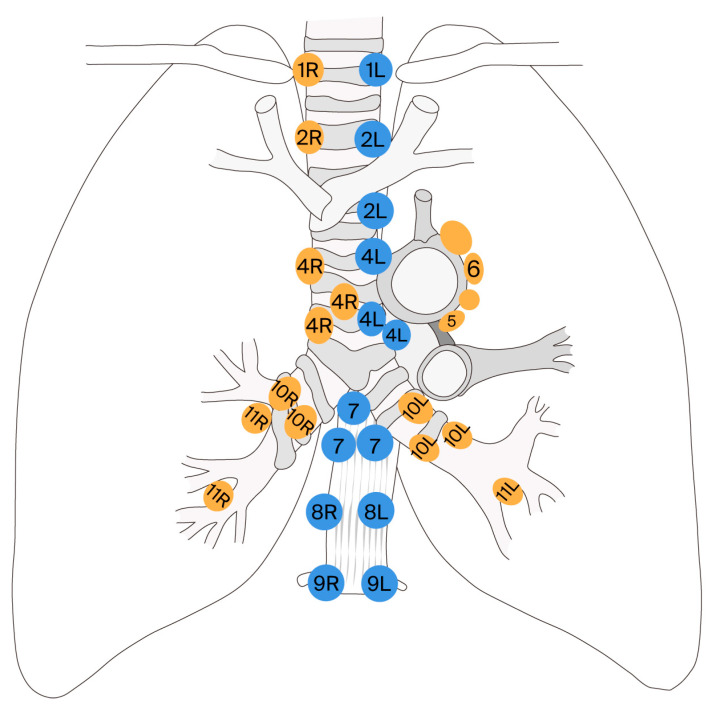
The different thoracic lymph node stations that can be approached by endoscopic ultrasonography. (Adapted after Kurt G. Tournoy et al. [29]).

In Level A staging, biopsies of every visible lymph node in all relevant stations (1, 2R, 2L, 3, 4R, 4L, and 7) are performed, requiring at least three passes through each lymph node or ROSE. Station 3A cannot be accessed through EBUS-TBNA due to the obstruction of large vessels in the mediastinum (Figure 2 [29]). Level B staging requires samples from stations 2R, 2L, 4R, 4L, and 7, using at least three passes per lymph node or ROSE. It is important to note that both Level A and Level B staging need to include samples from stations 5 and 6 if the tumor is in the left upper lobe, although these stations are generally inaccessible using EBUS-TBNA [30].

Level C staging corresponds to at least one adenopathy biopsied with fewer than three passes and no ROSE. The ability to freeze images allows for precise measurement of the evaluated nodule, ensuring accuracy in the assessment. The Doppler mode proves to be particularly valuable in evaluating vascularity within the ultrasound field, facilitating the safe insertion of the needle into the lymph node while adjusting for the 20-degree angle between the needle and transducer. Aspiration is performed by advancing the needle gently within the lymph node, with the aspirated material then transferred to glass slides for fixation and subsequent histopathological analysis. Also, material is collected for the preparation of cytoblocks or microbiological analysis, which may be essential for alternative or concurrent diagnostic evaluations [31]. Several retrospective studies have investigated the ultrasound characteristics of lymph nodes. In the most extensive study conducted by Fujiwara et al., a high negative predictive value for malignancy was observed in the absence of specific signs, including a round shape, well-defined margins, heterogeneous echogenicity, and the presence of coagulation necrosis [32]. Another study further corroborated that enlargement of the lymph node and its round or oval morphology are significant risk factors for malignancy. Increased vascularity within lymph nodes, especially beyond the main vessels leading to the center, can be an important indicator of malignant transformation. The absence of a central vessel in the lymph node during ultrasound evaluation is also recognized as a potential predictive sign of malignancy [33]. A 2008 study by Lee et al. highlighted the importance of the number of aspiration attempts on the diagnostic success rate of EBUS-TBNA. The same study found that performing three aspiration attempts resulted in a sensitivity of 95% and a specificity of 100%, which was significantly superior to the results observed with one or two aspiration attempts, where sensitivities were 69% and 83%, respectively. These findings emphasize the importance of multiple aspirations in enhancing the diagnostic accuracy of EBUS-TBNA [34]. A recent study in Toronto compared EBUS-TBNA with mediastinoscopy. All 153 eligible patients underwent EBUS-TBNA first, followed by mediastinoscopy, with both procedures showing similar results in evaluating the status of mediastinal lymph nodes. EBUS-TBNA exhibited a sensitivity of 81% and a specificity of 100%, while mediastinoscopy had a sensitivity of 79% and a specificity of 100% (Table 1).

Mediastinoscopy is an invasive surgical technique used in current practice for staging and diagnosing NSCLC. This method allows for direct visualization of mediastinal structures and biopsy of mediastinal lymph nodes, offering definitive histopathological confirmation, essential for determining the appropriate treatment strategy. While imaging methods like CT or PET-CT guide initial staging, mediastinoscopy remains the gold standard, especially in situations where non-invasive techniques such as EBUS-TBNA yield inconclusive results. Its fundamental role in identifying N2/N3 nodal involvement will have a significant impact on therapeutic decisions, being essential for establishing the optimal therapeutic regimen and improving the chances of patient survival [35]. Given the known less invasive nature of EBUS-TBNA, choosing this procedure instead of mediastinoscopy is justified, but the final decision depends on the physician’s experience and the medical institution’s available equipment [36].

However, certain clinical and radiological scenarios may necessitate complementary imaging methods, particularly for accurate staging, such as PET-CT. PET/CT plays an essential role in the diagnosis and staging of NSCLC and provides both anatomical information (via CT) and functional insights (via PET) [37,38]. This method is considered superior to other imaging techniques in early and accurate detection of malignant lesions, but it is not without potential confounding factors in the interpretation of the results. The use of the radiotracer 18F-FDG allows for the identification of regions with increased glucose metabolism, which is typical of tumor tissue [39]. Therefore, PET/CT is particularly useful for differentiating between benign and malignant nodules, especially in cases where conventional CT fails to offer definitive information [40]. In staging, PET/CT contributes significantly by evaluating disease extent, detecting both nodal and distant metastases—factors that critically influence therapeutic planning. Furthermore, it can help avoid unnecessary surgical interventions in patients with advanced disease. However, PET/CT has certain limitations, including false positives in cases of inflammation or infection, as well as false negatives in tumors with low metabolic activity, such as mucinous adenocarcinomas [41]. If the tomographic appearance of a patient suspected of having NSCLC is manifested as a polypoid lesion, eccentric lumen narrowing, or circumferential wall thickening, a PET-CT should be performed to confirm the absence of mediastinal lymph node involvement and distant metastases [42]. Currently, in patients with T1 tumors or peripheral T2 tumors, most guidelines do not recommend performing invasive mediastinal staging in the case of normal-sized mediastinal lymph nodes without glucose uptake on PET-CT. On the other hand, lymph nodes with pathological fluorodeoxyglucose uptake on PET-CT should be confirmed histopathologically through EBUS-TBNA, or if that is not possible, via mediastinoscopy. Therefore, invasive mediastinal staging is indicated in patients with mediastinal adenopathies larger than 1 cm or hypermetabolic on PET-CT, central tumors, or N1 lymph node staging [43]. Although imaging modalities such as PET/CT provide valuable guidance when combined with flexible bronchoscopy in staging strategies, histopathological confirmation remains the diagnostic gold standard for NSCLC.

## 5. Emerging Technologies: AI and Bronchoscopic Innovation

Recent advances in robotics and artificial intelligence (AI) are reshaping bronchoscopic practice and NSCLC staging. These techniques are formulated to improve diagnostic accuracy, increase workflow efficiency, and expand procedural coverage. Thus, AI-based systems are being developed to aid in real-time interpretation of endoscopic and ultrasound images during bronchoscopic scanning and EBUS-TBNA scanning. These platforms can detect abnormal mucosal patterns, differentiate between malignant and benign nodules, and support automated navigation to optimal biopsy sites. Convolutional neural networks, a subset of deep learning models, have demonstrated diagnostic accuracy comparable to that of expert bronchoscopists in preliminary studies. Although promising, such systems require extensive validation and clinical integration [44]. Robotic-assisted bronchoscopy platforms provide adequate stability and access to peripheral lesions. Electromagnetic navigation bronchoscopy (ENB) allows for preplanned navigation to nodules with greater precision. These methods allow access to lesions smaller than 2 cm, especially those located outside the radius of standard bronchoscopy, and have a lower risk of pneumothorax compared with transthoracic needle biopsy. The combination with real-time CT imaging further improves targeting capabilities. Despite their potential, these technologies are largely limited to clinical trials or tertiary referral centers [45].

## 6. Limitations

This narrative review comprises a comprehensive overview of FB and its role in the staging of NSCLC. However, several inherent limitations must be emphasized that may affect the validity and generalizability of the findings. Firstly, the narrative format does not follow a systematic methodology for a literature search, inclusion, and critical appraisal. As a result, selection bias may be introduced, with the potential omission of relevant studies or overrepresentation of certain perspectives. Secondly, the lack of quantitative synthesis prevents the assessment of pooled diagnostic performance metrics such as sensitivity, specificity, and predictive values across multiple studies. Without a meta-analytical approach, the relative efficacy of advanced FB techniques—particularly EBUS-TBNA—cannot be precisely compared to alternative staging modalities such as mediastinoscopy or PET-CT. Moreover, the review may not fully capture the variability in clinical outcomes associated with operator experience, institutional protocols, or patient-specific factors, which are crucial in real-world applications. Additionally, potential complications, limitations in access to advanced technologies, and cost-effectiveness considerations are only briefly addressed. Finally, given the rapid evolution of diagnostic tools and lung cancer management strategies, some of the included evidence may become outdated, and developing technologies like robotic bronchoscopy or artificial intelligence-assisted image analysis are not comprehensively discussed.

## 7. Conclusions

This review examines the evolving role of minimally invasive methods (FB and EBUS-TBNA) in NSCLC staging. By showing their clinical value, diagnostic yield, and safety profiles, this compares them to imaging techniques like PET/CT and conventional methods like mediastinoscopy. PET/CT informs staging approaches, but histopathological validation is key and EBUS-TBNA has an edge in some situations. The article also explores emerging technologies, including artificial intelligence and robotic bronchoscopy, which are expected to enhance diagnostic accuracy in the future. In summary, this review provides a structured evidence-based framework for integrating endobronchial instruments in NSCLC staging to enable personalized decision-making.

## Figures and Tables

**Table 1:** Lymph node station identification: EBUS vs. mediastinoscopy.

Lymph Node Station	Name/Location	Accessible via EBUS	Accessible via Mediastinoscopy
1	Lower cervical/ supraclavicular lymph nodes	yes	no (generally not accessible)
2R/2L	Upper paratracheal (right/left)	yes	yes
3A	Pre-vascular	yes (difficult)	no
3P	Retrotracheal	yes (very rare)	no
4R/4L	Lower paratracheal (right/left)	yes	yes
5	Subaortic (aorto-pulmonary)	no	yes (only via extended mediastinoscopy)
6	Para-aortic/ anterior to pulmonary artery ligament	no	no
7	Subcarinal	yes	yes
8	Lower paraesophageal	yes	no
9	Pulmonary ligament	yes (very rare)	no
10R/10L	Hilar (right/left)	yes	yes (difficult)
11R/11L	Interlobar	yes	no

## Data Availability

The data presented in this study are available upon request from the corresponding author.

## Abbreviations

The following abbreviations are used in this manuscript:

NSCLC	Non-small-cell lung cancer
FB	Flexible bronchoscopy
EBUS-TBNA	Endobronchial ultrasound with fine-needle aspiration
PET-CT	Positron emission tomography
LC	Lung cancer
FDG	Fluorodeoxyglucose
AFB	Autofluorescence bronchoscopy
NBI	Narrow-band imaging
BAL	Bronchoalveolar lavage
WLB	White light bronchoscopy
ACCP	American College of Chest Physicians
EGFR	Epidermal growth factor
ALK	Receptor anaplastic lymphoma kinase
PD-L1	Programmed death-ligand 1
ROSE	Rapid on-site evaluation
AI	Artificial intelligence
ENB	Electromagnetic navigation bronchoscopy
